# Updating Indoor Air Quality (IAQ) Assessment Screening Levels with Machine Learning Models

**DOI:** 10.3390/ijerph19095724

**Published:** 2022-05-08

**Authors:** Ling-Tim Wong, Kwok-Wai Mui, Tsz-Wun Tsang

**Affiliations:** Department of Building Environment and Energy Engineering, The Hong Kong Polytechnic University, Hung Hom, Hong Kong; ling-tim.wong@polyu.edu.hk (L.-T.W.); hayley.tsang@polyu.edu.hk (T.-W.T.)

**Keywords:** machine learning model, indoor air quality (IAQ) index, screening, assessment

## Abstract

Indoor air quality (IAQ) standards have been evolving to improve the overall IAQ situation. To enhance the performances of IAQ screening models using surrogate parameters in identifying unsatisfactory IAQ, and to update the screening models such that they can apply to a new standard, a novel framework for the updating of screening levels, using machine learning methods, is proposed in this study. The classification models employed are Support Vector Machine (SVM) algorithm with different kernel functions (linear, polynomial, radial basis function (RBF) and sigmoid), k-Nearest Neighbors (kNN), Logistic Regression, Decision Tree (DT), Random Forest (RF) and Multilayer Perceptron Artificial Neural Network (MLP-ANN). With carefully selected model hyperparameters, the IAQ assessment made by the models achieved a mean test accuracy of 0.536–0.805 and a maximum test accuracy of 0.807–0.820, indicating that machine learning models are suitable for screening the unsatisfactory IAQ. Further to that, using the updated IAQ standard in Hong Kong as an example, the update of an IAQ screening model against a new IAQ standard was conducted by determining the relative impact ratio of the updated standard to the old standard. Relative impact ratios of 1.1–1.5 were estimated and the corresponding likelihood ratios in the updated scheme were found to be higher than expected due to the tightening of exposure levels in the updated scheme. The presented framework shows the feasibility of updating a machine learning IAQ model when a new standard is being adopted, which shall provide an ultimate method for IAQ assessment prediction that is compatible with all IAQ standards and exposure criteria.

## 1. Introduction

Indoor air quality (IAQ) has gained enormous attention in the past decade due to the considerable amount of time we spend indoors nowadays [1,2]. To tackle the problem of poor IAQ, different countries have their own set of IAQ standards, with different measurement parameters and range of exposure limits. Representative parameters, such as carbon dioxide (CO_2_) and respirable suspended particulates (RSP), are always on the list, while total volatile organic compounds (TVOC), carbon monoxide (CO), ozone (O_3_), formaldehyde (HCHO), airborne bacteria count (ABC) may be included, depending on the application purpose of the standard [3,4,5,6,7]. The exposure limits are usually established based on health risk analysis, in which lifelong exposure to that level of pollutant shall not produce significant adverse effects on the public [8].

Alternatively, instead of complying strictly with the IAQ standard, the screening approach for assessing IAQ has become popular in recent years due to its simplicity and cheaper monitoring cost. With a large enough sample size, we can find out the “common” IAQ problems one type of premises often experiences, therefore, identifying the representative IAQ parameters that explain the majority of poor IAQ. The simplest way to reduce the cost of IAQ assessment is to just measure these representative parameters and see if they exceed the standard. One of the most notable examples is using CO_2_ level as an indicator of acceptable IAQ to adjust the fresh air quantity [9]. However, this approach may overlook the possibility of having IAQ problems caused by other IAQ parameters; therefore, a surrogate approach was proposed to identify surrogate IAQ parameters that are not just representative but also statistically correlated with other IAQ parameters. An express assessment protocol using three or five IAQ parameters, developed by Hui et al. [10], successfully screened out more than 90% of offices with poor IAQ, which provided an alternative for IAQ pre-assessment without the need to conduct a full assessment (all nine parameters). This study gave insight into the ability of a limited number of parameters in identifying problematic IAQ. Further to that, Wong et al. [11] proposed using CO_2_, RSP and TVOC as the surrogate indicators for evaluating IAQ in offices. The dependence and the correlations of the other nine parameters on the levels of the proposed surrogate indicators were found to be statistically significant. The result served as strong support that CO_2_, RSP and TVOC could be good surrogate indicators for other IAQ parameters, in terms of representativeness, ease of measurement and the possibility of real-time monitoring [12]. Individually, CO_2_, RSP and TVOC represent occupant load and ventilation rate, system filtration performance and indoor activities, and emissions from building materials and finishes, respectively, which serve as good indicators for the general IAQ of an environment with a ventilation system. To sum up, using surrogate indicators for IAQ evaluation can reduce the scale of measurement, as some high-risk premises are already being screened out preliminarily, therefore, reducing the resources required to identify problematic premises [10,11].

Based on the aforementioned efforts for simplifying IAQ assessment, an efficient and cost-effective IAQ screening protocol was proposed by Wong et al. [13] for identifying asymptomatic IAQ problems. IAQ index, the average fractional dose to exposure limits of the representative pollutants, was proposed and was used to diagnose unsatisfied IAQ in air-conditioned offices in the study by Mui et al. [14]. IAQ indices from 525 offices were evaluated using a five-level screening test with thresholds determined by likelihood ratios of unsatisfactory IAQ. A likelihood ratio larger than 1 indicates a high-risk sample having an excessive occurrence of unsatisfactory IAQ, whereas a smaller than 1 likelihood ratio identifies a low-risk sample. Given the pre-test probability of unsatisfactory IAQ and the regional failure percentage of the Hong Kong IAQ Certification Scheme, the post-test probability of offices with unsatisfactory IAQ can be estimated using the IAQ screening test. This screening test with representative IAQ parameters provides a much simpler and cost-effective alternative for IAQ assessment. If an environment “fails” in the screening test (i.e., any one of the three surrogate indicators exceeds the exposure limit), immediate remedies can be decided on to improve the IAQ. If not, based on the post-test probability given by the screening test, facility management can determine the threshold of the test and threshold of the remedy regarding the willingness to invest manpower and resources in improving the IAQ. Further test, a comprehensive one, will only be needed if the screening test result is in between the two thresholds [14].

It is noteworthy that this approach does not simply test some of the parameters against the standard, but rather uses these parameters to predict the probability of dissatisfying the standard based on correlation. Therefore, an assessment model developed based on the levels of surrogate parameters and probability of failing an IAQ standard is essential in IAQ screening practice. More improvements have been made to the IAQ index to further reduce the resources required for IAQ screening [15]; however, as powerful as it is in screening the IAQ of similar environments, prior knowledge of the IAQ of premises in the region is required [10], and the index may not be applicable to other kinds of space or against another set of IAQ standards.

In fact, throughout the development of IAQ policy, exposure limits have been updated from time to time, based on collective professional judgement and managerial decisions with a balance of social acceptance. The World Health Organization (WHO) has been making constant efforts to improve and refine the air quality standards, since the establishment of the air quality guidelines on selected pollutants in 2005 [16], which include the REVIHAAP project to review the health impacts of air pollution [17], and the HRAPIE project to identify dose–response relationship for RSP, O_3_ and nitrogen dioxide (NO_2_) [18]. Results from these two projects supported the comprehensive review of the European Union air quality policy in 2013 and many follow-up consultations and discussion forums on the preparation for an updated guideline [19]. In September 2021, the WHO issued the new Global Air Quality Guideline that reduced levels of key air pollutants to address the accumulated pieces of evidence of health effects and significant risks associated with poor air quality [20]. In 2019, the IAQ standard in Hong Kong was updated with stricter exposure limits to meet the updated IAQ guidelines published by the World Health Organization. The update consisted of the removal of three comfort parameters, the inclusion of visual inspection of mould condition and more stringent limits for CO, RSP and radon (Rn). Considering that the IAQ index itself, the screening levels and the likelihood ratios were all developed using the old standard, it is essential to identify the effect of the new IAQ standard on the suitability and performance of the established screening methods and to provide a framework for “updating” the screening levels.

With exposure standards being updated regularly in practical situations without the quantitatively assessed probable impact of the tightening of levels, fine tuning the IAQ screening baseline is deemed necessary. However, given that past data were assessed using the old standard, the iterative process for baseline determination using newly collected data takes a long time and is not ideal for responding to the rapid change in the need for environmental control. This presents a problem if the standard is being updated. Can the existing IAQ assessment model based on a statistical analysis of old data be useful against the new standard?

In this study, we proposed using machine learning methods for the development of a surrogate IAQ assessment model, which may be a solution to the problem of an updated IAQ standard and avoid the iterative process for baseline determination. Machine learning is a state-of-the-art method for environmental prediction. It is commonly used in outdoor pollution predictions [21] and indoor energy simulations [22]. The awareness and application of machine learning modeling in IAQ emerged in the past decade. A comprehensive review of existing machine learning and statistical models for IAQ prediction, conducted by Wei et al. [23], suggested that the majority of existing research focuses on using machine learning algorithms to predict pollutant concentrations. The most popular statistical models applied to IAQ consist of artificial neural network (ANN), multiple linear regression (MLR), partial least squares (PLS), and random forest (RF). They focus on predicting the concentrations of airborne particles, including RSP, e.g., [24,25,26], CO_2,_ e.g., [27,28], NO_2_, e.g., [29] and Rn, e.g., [30,31], in indoor environments using outdoor data. Recently, the forecasting of IAQ has become popular for the sake of improving public health and well-being, since precautionary actions can be acted on ahead of time [32]. Machine learning methods, such as linear and non-linear autoregressive models [33], are used to develop IAQ forecasting models using the historical profile of IAQ parameters. As continuous monitoring of IAQ is required as the basis of time-series machine learning models, it is common to forecast temperature, e.g., [34,35], relative humidity, e.g., [35,36], CO_2_, e.g., [34,35,36] and CO, e.g., [36], as they can be easily monitored using low-cost sensors [23]. Forecasting the concentration of indoor aldehydes, volatile organic compounds (VOC), and semi-VOC using statistical models remains scarce [33], and an example of using the nonlinear threshold autoregressive (TAR) model and Chaos-dynamics-based model to forecast HCHO is presented in the study by Ouaret et al. [37]. All things considered, it is advisable to test and compare different statistical models for each specific case, as demonstrated by many studies that used machine learning methods for IAQ modelling [33].

Besides indoor air pollutant prediction and forecasting, there are other examples of applying machine learning methods in IAQ-related research that can be found in the literature. Zimmerman et al. [38] applied random forests (RFs) to improve low-cost sensor performance for more accurate IAQ monitoring. Leong et al. [39] used a support vector machine (SVM) for the prediction of the air pollution index (API) in Malaysia. Their study demonstrated that the radial basis function (RBF) kernel function could accurately and effectively predict API. Sarkhosh et al. [40] used a decision tree (DT) model to identify the most influential parameters that contributed to the prevalence of Sick Building Syndrome (SBS) in office buildings. The high prevalence of SBS was found to be related to job satisfaction, ergonomic parameters, microbiological pollutants and 1-methyl-4-(1-methylethyl) benzene concentration.

While IAQ prediction and forecasting give us a better understanding of the IAQ situation we are experiencing, it is of equal importance to identify whether the level of IAQ is considered acceptable or not before any follow-up mitigation or precautionary strategies are taken; therefore, an IAQ assessment model is essential.

To our best knowledge, we have identified the following research gaps in the field:

- Using machine learning methods to assess whether the IAQ is acceptable or not with a given IAQ standard;
- Addressing the issues of updating/changing IAQ standards, which would affect the screening levels and results; and
- Predicting the updated screening baselines of IAQ with new standards.

Therefore, in this study, we discuss the possibility of using machine learning methods to “update” the screening levels, such that the IAQ screening method can still be applicable with a new standard. Using Hong Kong’s case of an updated IAQ standard as an example, in this paper, we present a universal framework of using machine learning models in predicting the updated IAQ screening levels, which includes:

- Developing and evaluating the performance of machine learning IAQ assessment models with surrogate IAQ parameters;
- Quantifying the impact of an updated scheme (i.e., an IAQ standard) on the machine learning IAQ assessment model; and
- Evaluating the model flexibility in adapting an updated/another exposure standard.

Applicable to all IAQ standards and guidelines, this framework not only enables the implementation of a territory-wide IAQ screening program but also facilitates IAQ monitoring and improvements.

## 2. Materials and Methods

In the following section, the framework for updating the screening levels of IAQ assessment models is presented. To demonstrate the updating process, machine learning models for IAQ assessment based on the developed IAQ index algorithm and screening methodology were first developed using selected machine learning modelling methods. The performances of the models were evaluated, and with the average assessment results from the models, the relative impact ratios of the updated standard on the old standard were determined. The framework details the feasibility of developing machine learning IAQ assessment models, methods for model performance evaluation and the procedures for updating the screening levels with an updated standard.

### 2.1. Overview of the Data

IAQ assessment data collected from a cross-sectional IAQ survey of 525 air-conditioned offices in Hong Kong reported in a previous study was adopted to evaluate the performance of machine learning models [14]. The surveyed premises, which covered various grades, types and ages, included a wide range of open-plan offices from 10 m^2^ to 300 m^2^. The IAQ survey was conducted for the fulfilment of the Hong Kong IAQ Certification Scheme (the Scheme); therefore, the measurement protocol, sampling locations, period and equipment strictly followed the requirements stated in the Scheme. As such, 8 h continuous samplings were conducted during the office-occupied hours with a sampling density of 500 m^2^. All the sampling points were selected by the IAQ professionals during the walkthrough inspection before the actual measurement.

Two IAQ assessment schemes, Schemes 1 and 2, are exhibited in Table 1. Scheme 1 was the old IAQ objective in the Hong Kong IAQ Certification Scheme and Scheme 2 was the updated one to update the requirement against the latest IAQ guidelines by the World Health Organization [41]. In the updated scheme, exposure limits of CO, Rn and RSP are tightened to provide better public health protection. As mentioned above, the IAQ index using likelihood ratio cannot adapt to an updated standard since it was developed based on the previous standard, so using machine learning algorithms to model the IAQ index and IAQ dissatisfaction can, therefore, be a universal solution to the existing barrier.

A statistical summary of the dataset extracted for this study, which consists of three independent yet closely correlated IAQ surrogate indicators concerning the IAQ index [14], namely CO_2_, RSP and TVOC, is presented in Table 2. These three parameters were selected as the surrogate indicators among the remaining 9 pollutants in the Scheme, among which, RSP represents the filtering efficiency of the air-conditioning system, CO_2_ represents the occupant load and ventilation rate, and TVOC indicates building emission [13]. The overall summary of the dataset is shown at the top of the table, with the range of CO_2_ = 339–1497 ppm, RSP = 4–125 μg m^−3^, TVOC = 0–3144 μg m^−3^ and the calculated IAQ index = 0.189–1.99. Using the two assessment schemes introduced in Table 1 above, this dataset was further classified into “Satisfactory IAQ” (i.e., if all of the 9 pollutant levels fulfil the assessment scheme) or “Unsatisfactory IAQ” (i.e., 1 or more of the 9 pollutant levels fail the assessment scheme). While the mean values of CO_2_, RSP and TVOC in the “Satisfactory IAQ” group were significantly different from those in the “Unsatisfactory IAQ” group (*p* < 0.05, *t*-test), the sample (satisfactory or unsatisfactory) group means results from Schemes 1 and 2 were statistically the same (*p* > 0.1, *t*-test). Table 2 also exhibits the IAQ index *θ*, which is an IAQ indicator determined using Equation (1), with *j* = 1,…,3, Φ_j_* being the fractional dose of RSP, CO_2_ and TVOC, *Φ_j_* the exposure level of the assessed parameter over an exposure time, and *Φ_j,e_* the reference exposure limit under Scheme 1 (RSP = 180 μg m^−3^, CO_2_ = 1000 ppm, TVOC = 600 μg m^−3^) [15].

(1) $$\theta =\frac{1}{3}\sum_{j=1}^{3}\Phi_{j}^{*};\;\Phi_{j}^{*}=\frac{\Phi_{j}}{\Phi_{j,e}}$$

### 2.2. Data Preprocessing

**Figure 1:**
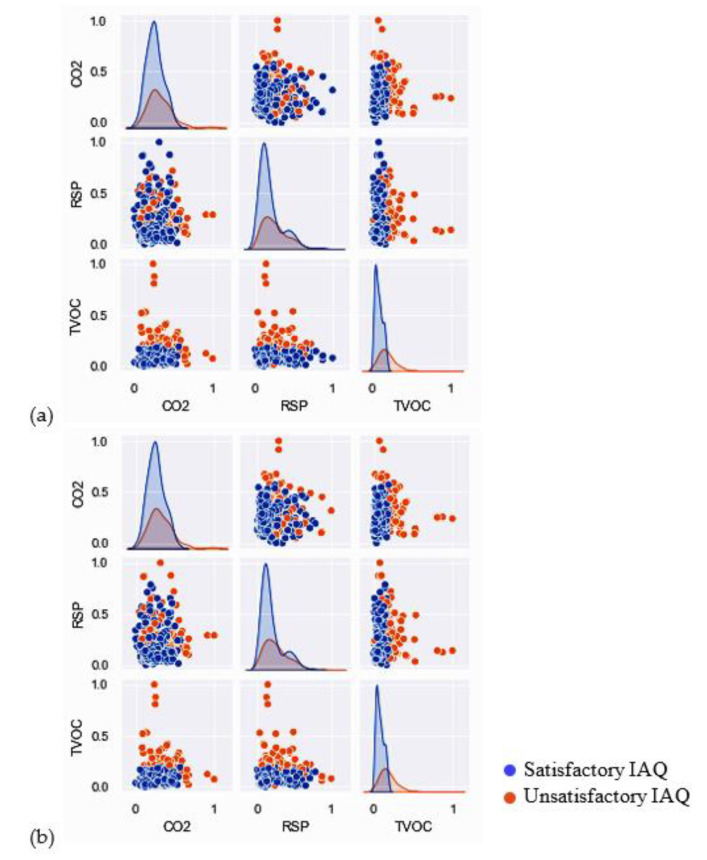
Pair plots of CO_2_, RSP, and TVOC grouped by assessed indoor air quality (IAQ) against assessment (**a**) Scheme 1 (**b**) Scheme 2.

Figure 1 shows the pair plots of the IAQ parameters grouped by satisfactory and unsatisfactory IAQ assessed using Schemes 1 and 2. A linear data scaling to the range [0, 1] was applied for data normalization.

The training data and testing data were randomly selected at a distribution ratio of training data (1 − *r_d_*) and testing data (*r_d_*), as shown in Equation (2), where *n_d_*_,*t*_ and *n_d_*_,*g*_ are the numbers of data points in the testing and training datasets, respectively.

(2) $$r_{d}=\frac{n_{d,t}}{n_{d,g}}$$

Multifold cross-validation was employed for model validation. The training dataset was divided into 5 and 10 subsets of equal size and each subset was tested using the hyperparameters trained on the remaining subsets. The cross-validation accuracy was determined based on the percentage of correctly classified data. A grid search was then conducted to optimize the model hyperparameters, which were later used to retrain the model for evaluation.

The model accuracy *AC*, the probability of the model making a correct prediction [14], is usually compared with the baseline accuracy *AC_bl_* in Equation (3) which indicates the certainty of the predictions made without the algorithm, where mode (*N*) is the mode of true result and *N* is the sample size.

(3) $$AC_{bl}=\frac{\mathrm{mode}\;\left(N\right)}{N}$$

The baseline accuracy values adopted are 0.682 and 0.670 for Schemes 1 and 2, respectively. A model with an accuracy below the baseline is considered to be unsatisfactory.

**Figure 2:**
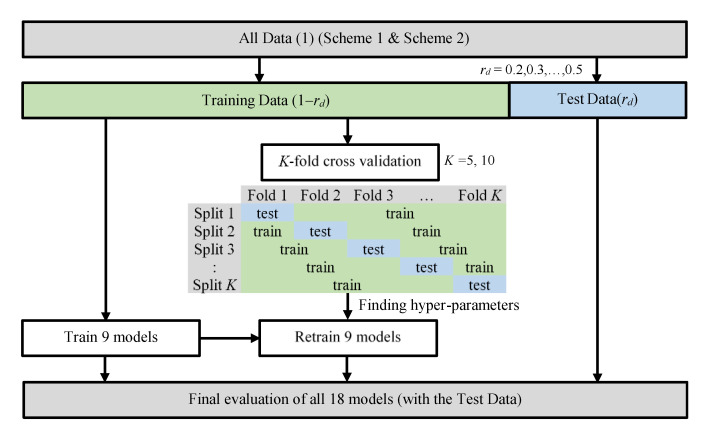
Data processing for model training and evaluation.

In this study, as shown in Figure 2, a total of 16 (=4 × 2 × 2) evaluation conditions were generated from 4 different combinations (*r_d_* = 0.2, 0.3, 0.4, 0.5) of training and testing data, 2 multifold cross-validations (*K* = 5, 10) and 2 IAQ schemes (Schemes 1 and 2). Trained models (without grid-search-tuned model hyperparameters) and retrained models (with grid-search-tuned model hyperparameters) were then evaluated using the testing data of the 16 evaluation conditions, and finally, 32 sets of testing results were obtained for evaluating the performance of the 9 models for IAQ assessment.

### 2.3. Models for Evaluation

Table 3 shows the classification models (classifiers) employed for developing the IAQ assessment model. The selected models included Support Vector Machine (SVM) with different kernel functions (i.e., linear, polynomial, radial basis function (RBF), and sigmoid), k-Nearest Neighbors (kNN), Logistic Regression, Decision Tree (DT), Random Forest (RF) and Multilayer Perceptron Artificial Neural Network (MLP-ANN). These algorithms are commonly used for developing IAQ prediction and forecasting models based on the literature review described in the introduction. In order to provide a universal framework for developing the IAQ assessment models and updating the screening levels, these popular models were adopted and their performances were evaluated. More details of each machine learning model and its hyperparameters can be found in Appendix A.

Table 3 also presents the test ranges of the hyperparameters, the cross-validation accuracy and the model accuracy with the testing datasets, and the corresponding hyperparameters that gave the best prediction accuracy in all tests. The development and the training of models were coded using the Python programming language described by Pedregosa et al. [42].

Regularization was applied to avoid overfitting by penalizing large coefficients [43]. It was intended to reduce the generalization error but not the training error. As a result, the application of regularization allowed a certain amount of misclassified data points in the training dataset [44]. To minimize the error between the true value *y_i_* and the predicted value *xβ*, the cost function *f* shown in Equation (4) could be expressed with the L2 loss function $\underset{i}{\sum }{\left(y_{i}-\underset{j}{\sum }x_{ij}\beta_{j}\right)}^{2}$ and the regularization factor *C* [45].

(4) $$f=\underset{i}{\sum }{\left(y_{i}-\underset{j}{\sum }x_{ij}\beta_{j}\right)}^{2}+C\underset{j}{\sum }\beta_{j}^{2}$$

## 3. Results and Discussion

**Figure 3:**
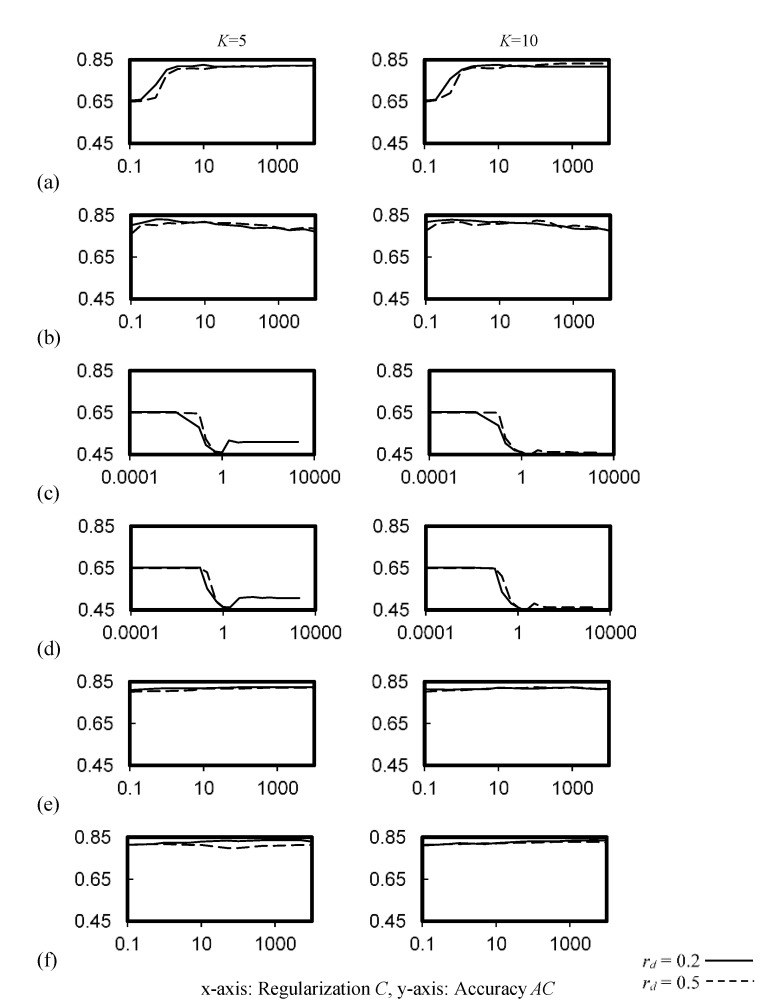
Cross-validation accuracy of the SVM classifier. (**a**) Linear kernel, (**b**) rbf kernel, (**c**) sigmoid kernel, *c*_0_ = 0.01, (**d**) sigmoid kernel, *c*_0_ = 0.5, (**e**) polynomial kernel, *c*_0_ = 0, *c*_1_ = 2, (**f**) polynomial kernel, *c*_0_ = 1, *c*_1_ = 3.

Figure 3 illustrates the cross-validation accuracy of the SVM classifiers with linear, RBF, sigmoid and polynomial kernels. Consistent accuracy of *AC* > 0.8 was observed when the regularization factor *C* was ≥2 for the SVM with linear kernel, and for the whole test ranges of the SVM with RBF and polynomial kernels. However, the SVM with sigmoid kernel did not perform well for the training datasets, as compared with other kernels, with *AC* ≤ 0.65, which dropped significantly for *C* ≥ 0.6.

**Figure 4:**
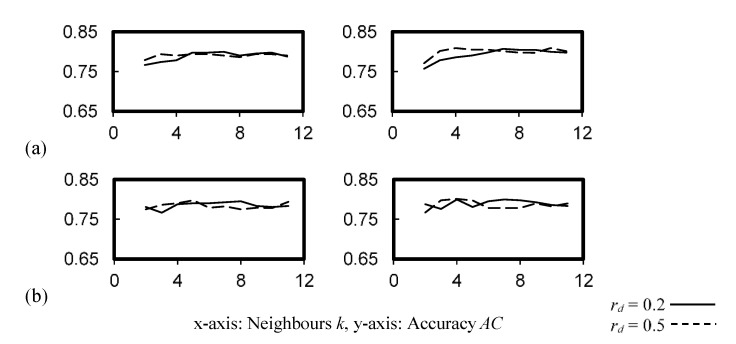
Cross-validation accuracy of the *k*NN classifier. (**a**) *W* = 1/*d_k_*, (**b**) *W* = 1.

Figure 4 shows the cross-validation accuracy of the *k*NN classifier, which was consistent for *k* = 2–11. While the accuracy was more sensitive to the weight function applied, a larger *k* that compensated for the accuracy drop was observed in Figure 4a.

**Figure 5:**
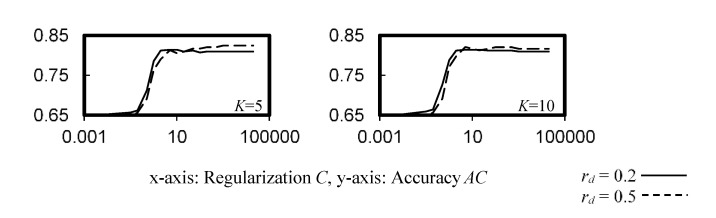
Cross-validation accuracy of the logistic classifier.

According to Figure 5, the logistic regression classifier improved the prediction accuracy for regularization factor *C* > 2. The choice of training dataset was found to be insignificant to the model accuracy.

**Figure 6:**
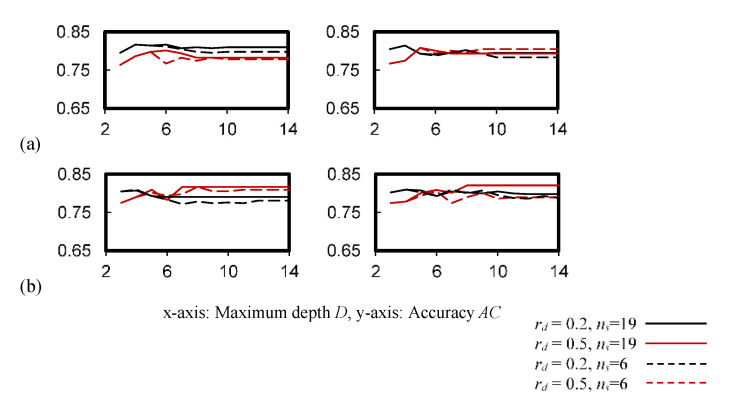
Cross-validation accuracy of the decision tree classifier. (**a**) Entropy impurity, *n_r_* = 6 (**b**) Gini impurity, *n_r_* = 2.

Figure 6 graphs the cross-validation accuracy of the decision tree classifier. Within the range of 0.75–0.8, the accuracy was sensitive to the size of the dataset, the impurity function, the minimum number of samples required to split an internal node *n_s_*, and the minimum number of samples required to be at a leaf node *n_r_*. It became less sensitive when the maximum depth value was greater than or equal to 10 (i.e., *D* ≥ 10).

**Figure 7:**
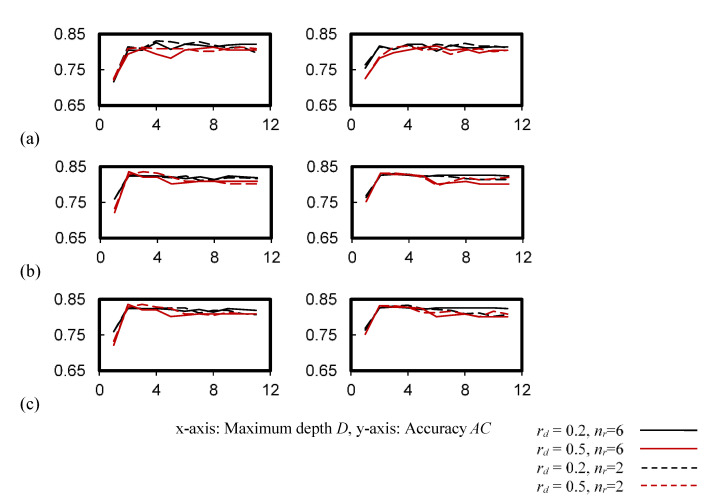
Cross-validation accuracy of the random forest classifier. (**a**) Entropy impurity, *n_s_* = 9, *n_f_* = 10 (**b**) Gini impurity, *n_s_* = 9, *n_f_* = 110, (**c**) Gini impurity, *n_s_* = 2, *n_f_* = 110.

Figure 7 exhibits the cross-validation accuracy of the random forest classifier. The accuracy, which became less sensitive for *D* ≥ 2, was improved, as compared with Figure 6. It can be seen that the number of trees *n_f_* compensated for the accuracy drop due to *D* ≤ 5.

**Figure 8:**
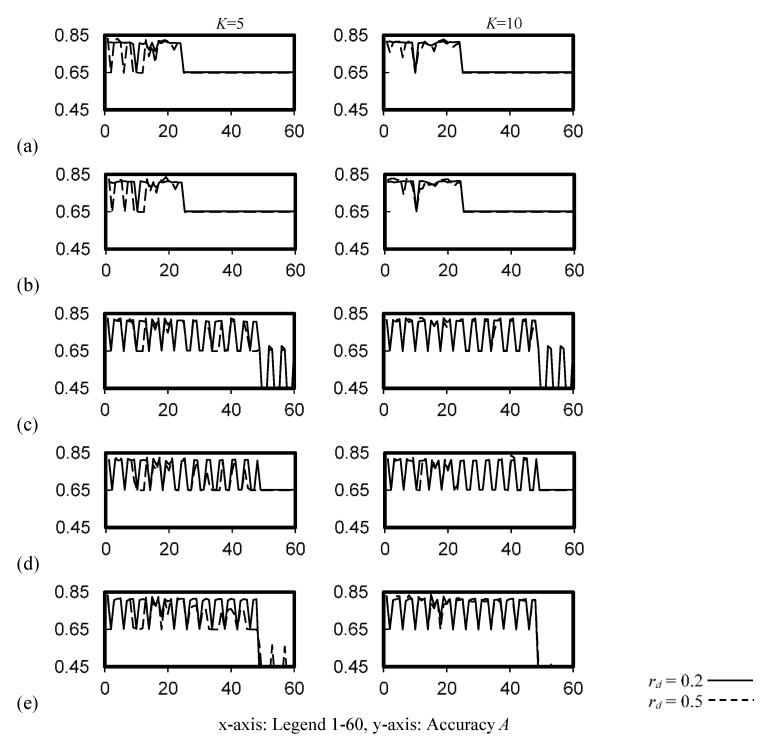
Cross-validation accuracy of the MLP-ANN classifier. (**a**) 100 neurons, 1 hidden layer, (**b**) 200 neurons, 1 hidden layer, (**c**) 100 neurons, 6 hidden layers (**d**) 200 neurons, 6 hidden layers, (**e**) 100 neurons, 3 hidden layers.

A wide range of hyperparameters can be adopted for a MLP-ANN classifier. In this study, 100 and 200 neurons in the inner layers 1, 3, 4 and 6 were evaluated, with neuron arrangements of each layer in the ratios of (1), (1:8:1), (1:4:4:1) and (1:2:2:2:2:1). Figure 8 illustrates the cross-validation accuracy of the 60 configurations of the model hyperparameters for the inner-layer architecture (i.e., *x*-axis with legends 1–60, Table A1). A very sensitive accuracy ranging from <0.45 to about 0.8 was observed.

It was challenging to set up a suitable MLP-ANN for an engineering application without prior selection of the model hyperparameters. Table 4 shows the test accuracy of the MLP-ANN classifier. The identity activation function made the best predictions with the highest (mean and median) test accuracy. Iteration schemes ADAM and L-BFGS, with constant learning rates only, returned more accurate predictions, as compared with SGD.

To sum up, all of the IAQ assessment models developed achieved the maximum test accuracy, in a narrow range of 0.807–0.820, with the mean test accuracy ranging from 0.536 to 0.805. Table 5 presents the best-performed models in the 32 tests (16 each for the trained and retrained models). The results showed that the SVM with polynomial kernel gave the highest test accuracy and next-best predictions in the trained and retrained model tests. Moreover, models with decision tree and random forest classifiers gained 4 and 3 counts (out of 16), respectively, in the trained model test, whereas the SVM with linear kernel gained 8 counts (i.e., the best prediction performance) in the retrained model test. These classifiers can be good choices for accurate IAQ assessment model development.

## 4. Model Prediction of IAQ Assessment with IAQ Index Updates

The IAQ index was developed previously as a screening strategy to screen out premises with problematic IAQ based on assessment Scheme 1. Given that the assessment scheme has been updated to Scheme 2, this section evaluates the relative impact of the index due to the updated values of baselines in the two schemes.

The relative impact on the IAQ index for IAQ assessment with Schemes 1 and 2 was evaluated using three uniformly distributed ranges: CO_2_ = 400–1400 ppm, RSP = 1–120 μg m^−3^, and TVOC = 0–1500 μg m^−3^. The selected ranges of surrogate pollutants generally cover the observable range in the office IAQ database. Determined by Monte Carlo sampling techniques, the three IAQ parameters in the above ranges were used to calculate the corresponding IAQ index and to predict the IAQ satisfaction/dissatisfaction using the trained and retrained classifiers.

**Figure 9:**
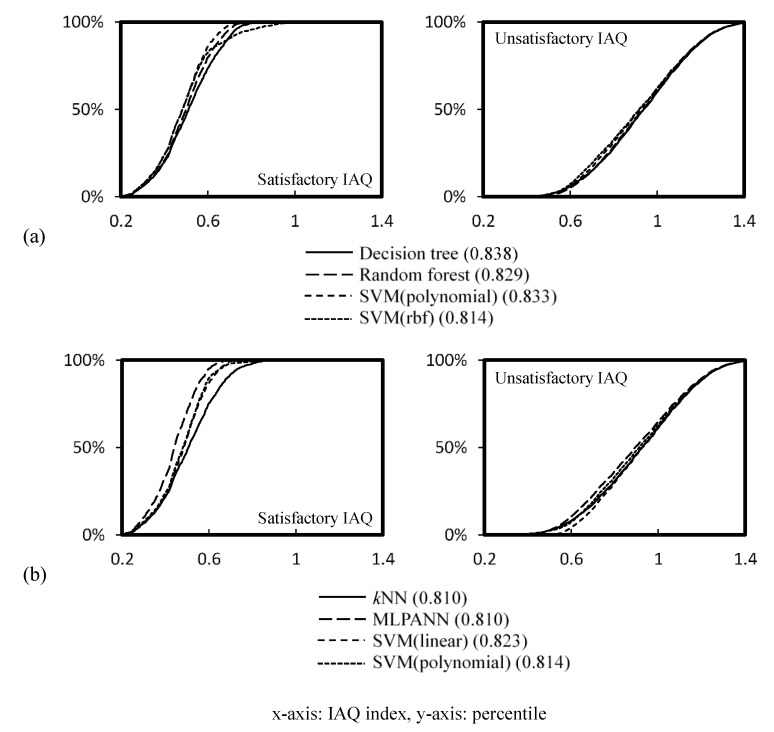
Predicted IAQ satisfaction and dissatisfaction with an IAQ index with assessment criteria, (**a**) Scheme 1, (**b**) Scheme 2.

Figure 9 shows the percentage of predicted satisfactory and unsatisfactory IAQ for the range of IAQ indices under Schemes 1 and 2. The IAQ satisfaction was assessed by the best performing trained and retrained IAQ classification models (with model accuracy shown in brackets). Classifications were performed with models with classifiers of a decision tree, a random forest, SVM with polynomial kernel and RBF kernel for Scheme 1, and models with classifiers of *k*NN, MLP-ANN, SVM with linear kernel and polynomial kernel for Scheme 2. The figure shows that the predictions of unsatisfactory IAQ made by these models generally agree with each other, with a deviation up to ±5% from the average prediction of satisfactory IAQ with Scheme 2.

The IAQ index in Figure 9 does not map any particular office distribution function and, thus, a relative approach was adopted to study the relative impact of Scheme 2 on Scheme 1, in terms of assessment likelihood, using the dataset summarized in Table 2. The relative impact ratio *r*_2,1_ is determined by Equation (5), where *x_u_* and *x_s_* are the distribution functions of the IAQ index for unsatisfactory and satisfactory IAQ respectively.

(5) $$r_{2,1}=\frac{LR_{2}}{LR_{1}};\;LR=\frac{\int_{x_{1}}^{x_{2}}f\left(x_{u}\right)dx}{\int_{x_{1}}^{x_{2}}f\left(x_{s}\right)dx}$$

Table 6 outlines a proposed likelihood ratio *LR*_1_ for air-conditioned offices with unsatisfactory IAQ using Scheme 1, as reported in an earlier study [29]. The estimation of *r*_1,2_ was made based on the average predictions from all models shown in Figure 9. Normality of the IAQ index was assumed (*p* > 0.05, *w*/*s* test). Based on the relative impact values determined for the IAQ index ranges <0.32, 0.32–0.42, 0.43–0.53, 0.54–0.64, ≥0.65, the corresponding values of *LR*_2_ were computed (by *LR*_2_ = *r*_2,1_ *LR*_1_) and summarized in Table 6. The corresponding likelihood ratios in Scheme 2 were found to be higher due to the tightening of assessment criteria in the updated scheme.

## 5. Conclusions

One of the ongoing IAQ development tasks is to constantly improve IAQ objectives so that they are updated, relevant and attainable. Territory-wide IAQ screening should be implemented immediately, and later, periodically, to understand the overall IAQ situation and to maintain an up-to-date IAQ profile. Given so many IAQ standards with a wide range of exposure limits established by various governments, a universal framework for IAQ assessment modelling, which applies to all standards, is of urgent need.

In this study, a new strategy for unsatisfactory IAQ prediction using machine learning models of three surrogate IAQ indicators in the IAQ index was proposed. The results showed that all selected machine learning models performed well, achieving a maximum test accuracy of 0.807–0.820. Among the selected models, SVM with linear kernel and polynomial kernel, decision tree classifier and random forest classifier gave an IAQ classification with higher accuracy. To further demonstrate the use of IAQ index with different exposure limits in IAQ assessment, machine learning models of IAQ index using two different baselines (Schemes 1 and 2) were presented. The predictions of IAQ made by all selected models generally agreed with each other, with a ±5% deviation observed in the prediction of satisfactory IAQ under Scheme 2. The likelihood ratio of the IAQ index in Scheme 2 also increased with the tightening criteria for assessing exposure levels.

As demonstrated, machine learning models for IAQ index give promising prediction accuracy in identifying unsatisfactory IAQ, and that shall provide an ultimate strategy for IAQ screening and assessment, even under various IAQ standards and exposure criteria.

## Figures and Tables

**Table 1:** 8 h exposure limits of satisfactory indoor air quality.

Parameter (Unit)	Scheme 1	Scheme 2
CO_2_ (ppm)	1000	1000
CO (ppm)	8.7	6.1
RSP (μg m^−3^)	180	100
NO_2_ (μg m^−3^)	150	150
O_3_ (μg m^−3^)	120	120
HCHO (μg m^−3^)	100	100
TVOC (μg m^−3^)	600	600
Radon (Bq m^−3^)	200	167
Airborne bacteria (CFU m^−3^)	1000	1000

**Table 2:** Statistical summary of levels of indoor air quality surrogate parameters in 525 offices, (**a**) overall summary; (**b**) summary of the dataset being classified as “Satisfactory IAQ” regarding Schemes 1 and 2; (**c**) summary of the dataset being classified as “Unsatisfactory IAQ” regarding Schemes 1 and 2.

**(a) Overall Summary**				
	**CO_2_ (ppm)**	**RSP (μg m^−^^3^)**	**TVOC (μg m^−3^)**	**IAQ Index**
mean	658	30	358	0.473
std dev	151	20	328	0.201
min	339	4	0	0.189
25%	556	15	140	0.333
50%	639	22	295	0.431
75%	746	38	466	0.558
max	1497	125	3144	1.99
**(b) Satisfactory IAQ**				
**Scheme 1**				
Count	358			
mean	634	28	242	0.397
std dev	126	20	152	0.111
min	339	4	0	0.189
25%	546	14	113	0.312
50%	624	20	209	0.381
75%	714	33	354	0.477
max	998	125	597	0.725
**Scheme 2**				
Count	352			
mean	634	27	240	0.394
std dev	126	18	152	0.110
min	339	4	0.0	0.189
25%	547	14	112	0.311
50%	623	20	208	0.378
75%	713	32	354	0.474
max	998	99	597	0.725
**(c) Unsatisfactory IAQ**				
**Scheme 1**				
Count	167			
mean	709	34	607	0.637
std dev	184	19	446	0.249
min	396	7	45	0.202
25%	384	19	346	0.488
50%	678	29	517	0.406
75%	807	44	738	0.737
max	1497	91	3144	1.991
**Scheme 2**				
Count	173			
mean	707	36	598	0.634
std dev	183	22	442	0.246
min	396	7	45.0	0.202
25%	583	19	338	0.487
50%	678	29	497	0.603
75%	804	46	715	0.725
max	1497	125	3144	1.991

**Table 3:** Selected machine learning models and hyperparameters for the development of IAQ assessment models.

Models	Hyper-Parameters	Test Range	Validation Accuracy	Test Accuracy	Hyperparameters Used
SVM (linear)	*r_d_* *C*	0.2–0.5 0.1–10,000	0.794–0.832	0.752–0.824	0.4 1.0
SVM (polynomial)	*r_d_* *C* *c*_1_ *c*_0_	0.2–0.5 0.1–10,000 2, 3 0, 1	0.813–0.839	0.753–0.833	0.4 1000 3 1
SVM (rbf)	*r_d_* *C*	0.2–0.5 0.1–10,000	0.806–0.831	0.762–0.824	0.4 1.0
SVM (sigmoid)	*r_d_* *C* *c*_0_	0.2–0.5 0.0001–2000 0–1	0.638–0.652	0.443–0.800	0.2 0.0001 0
*k*NN	*r_d_* *k* *W*	0.2–0.5 2, 3, …, 11 1, 1/*d_k_*	0.785–0.809	0.762–0.824	0.4 10 1
Logistic regression	*r_d_* *C*	0.2–0.5 0.001–20,000	0.790–0.825	0.753–0.810	0.4 1
Decision tree	*r_d_* *D* *n_s_* *n_r_* Impurity	0.2–0.5 3, 4, …, 14 3, 4, …, 19 2, 3, …, 6 *GI*, *EI*	0.805–0.829	0.714–0.838	0.2 4 3 2 *EI*
Random forest	*r_d_* *n_f_* *D* *n_s_* *n_r_* Impurity	0.2–0.5 10, 60, 110 1, 2, …, 11 1, 2, …, 9 2, 3, …, 6 *GI* or *EI*	0.824–0.844	0.724–0.829	0.3 60 2 3 1 *GI*
MLP-ANN	*r_d_* *C* Neurons Hidden layer Activation Iteration Learning rate	0.2–0.5 0.0001, 0.05, 1 100, 200 1, 3, 4, 6 Identity, logistic, *tanh*, relu LBFGS, SDG, Adam Constant, invscaling, adaptive	0.807–0.836	0.714–0.810	0.4 0.0001 200 3 relu LBFGS Constant

**Table 4:** Test accuracy of the MLP-ANN classifier (5-fold and 10-fold).

Hyper-Parameters			Test Accuracy			
Activation	Iteration	Learning Rate	Mean	Median	Min	Max
identity	All		0.740	0.795	0.336	0.836
logistic			0.636	0.646	0.348	0.828
*tanh*			0.728	0.783	0.348	0.836
relu			0.701	0.743	0.348	0.836
all	ADAM	Constant	0.765	0.801	0.638	0.832
	LBFGS	Constant	0.767	0.802	0.638	0.836
	SGD	Adaptive	0.712	0.648	0.638	0.828
		Constant	0.712	0.648	0.638	0.836
		invscaling	0.550	0.646	0.336	0.676
identity	ADAM	constant	0.801	0.806	0.641	0.832
	LBFGS	constant	0.805	0.806	0.778	0.824
	SGD	adaptive	0.758	0.793	0.638	0.828
		constant	0.758	0.791	0.638	0.836
		invscaling	0.579	0.646	0.336	0.668
logistic	ADAM	constant	0.667	0.646	0.638	0.828
	LBFGS	constant	0.683	0.646	0.638	0.820
	SGD	adaptive	0.646	0.646	0.638	0.652
		constant	0.646	0.646	0.638	0.652
		invscaling	0.536	0.646	0.348	0.652
relu	ADAM	constant	0.794	0.804	0.638	0.832
	LBFGS	constant	0.797	0.804	0.638	0.836
	SGD	adaptive	0.689	0.646	0.638	0.823
		constant	0.689	0.646	0.638	0.826
		invscaling	0.536	0.646	0.348	0.652
*tanh*	ADAM	constant	0.799	0.805	0.641	0.832
	LBFGS	constant	0.782	0.772	0.702	0.824
	SGD	adaptive	0.754	0.786	0.638	0.826
		constant	0.755	0.786	0.638	0.836
		invscaling	0.548	0.646	0.348	0.676

**Table 5:** The most accurate classifiers in 32 comparison tests.

Classifier	Trained Model		Retrained Model		Trained & Retrained Models	
	Count (*N* = 16)	Test Accuracy	Count (*N* = 16)	Test Accuracy	Count (*N* = 16)	Test Accuracy
SVM (linear)	0		8	0.811	8	0.811
SVM (polynomial)	6	0.820	6	0.816	12	0.818
SVM (rbf)	0		2	0.814	2	0.814
SVM (sigmoid)	0		0		0	
*k*NN	2	0.807	0		2	0.807
Logistic regression	0		0		0	
Decision tree	4	0.814	0		4	0.814
Random forest	3	0.819	0		3	0.819
MLP-ANN	1	0.810	0		1	0.810

**Table 6:** IAQ index of air-conditioned offices in Hong Kong.

IAQ Index θ	Likelihood Ratio (Scheme 1) *LR*_1_	Relative Impact *r*_2,1_	Likelihood Ratio (Scheme 2) *LR*_2_
<0.32	0.1	1.4	0.1
0.32–0.42	0.4	1.2	0.5
0.43–0.53	0.8	1.1	0.9
0.54–0.64	1.7	1.3	2.2
≥0.65	25	1.5	38

## Data Availability

Data available on request.

## Nomenclature

*IAQ index and updates*	
*j*	surrogate parameter
*Φ* *_j_* *^*^*	fractional dose
*Φ_j_*	exposure level
*Φ_j,e_*	reference exposure limit
*θ*	IAQ index
*r*	relative impact ratio
*x_u_/x_s_*	distribution functions for unsatisfactory/satisfactory IAQ index
*LR*	likelihood ratio
*Data processing*	*Data processing*
*X*	data vector
*r_d_*/1 − *r_d_*	test data/training data
*n_d,t_/n_d,g_*	number of data points in the test/training set
*AC*	model accuracy
*AC_bl_*	baseline accuracy
*TP/TN*	true positive/negative
*FP/FN*	false positive/negative
*N*	sample size
*K*	number of folds
*Units for IAQ parameters*	
ppm	parts per million
μg m^−3^	microgram per cubic meter
Bq m^−3^	becquerels per cubic meter
CFU m^−3^	colony-forming units per cubic meter
*Regularization*	
*f*	cost function
*y_i_*	true value
*x* *β*	predicted value
*C*	regularization factor
*n*	number of dimensions
*Decision tree/random forest*	
*p_j_^2^*	probability of *j*
*j*	class
*D*	tree’s maximum depth
*n_s_/n_r_*	minimum number of samples required to split an internal node/be at a leaf node
*n_f_*	number of trees
*Support Vector Machines*	
*α, β*	constants
*x_i_*	inputs
*y_i_*	output class
*M*	margin half-width
*ε_i_*	slack variables
*c_0_, c_1_*	hyperparameters for *K(x_i_,x_j_)*
*K(x_i_,x_j_)*	kernel function
*γ*	kernel coefficient
*k-Nearest Neighbors*	
*k*	constant
*d(x_i_,y_i_)*	Euclidean distance
$\hat{y}$	predictions
*W*	weight function
*d_k_^−1^*	neighbour distance
MLP-ANN	
*R*	dataset
*m/o*	dimension for input/output
*J*	local gradient of function f
*β*	parameter
*y*	independent variables
*δ*	increment
*Logistic regression*	
*x*_0_	sigmoid’s midpoint of *x*
*x*	inputs
*k*	logistic growth rate
*w*	coefficient vector

## Appendix A

### Appendix A.1. Support Vector Machine (SVM)

The support vector machine (SVM) algorithm identifies the optimal hyperplane in an *n*-dimensional space that distinctly separates the data points to be classified into two classes (in this study, satisfaction or dissatisfaction). The algorithm maximizes the margin between these two classes. The linear classifier can be expressed by Equation (A1), where *α* and *β* are constants, *x* is the input vector of inputs *x_i_* [46,47], and *y_i_* is the output class.

(A1) $$f\left(x\right)=\beta_{0}+\underset{i}{\sum }\alpha_{i}\langle x_{i},x\rangle ;\;f\left(y_{i}\right)=\left\{\begin{array}{cc} 0 & f\left(x_{i}\right)<0\\ 1 & f\left(x_{i}\right)>0 \end{array}\right.$$

To maximize the margin half-width *M* of the strip that separates the data points into the two classes, slack variables *ε_i_* are specified for the soft margins, such that observations (training data) on the wrong side are allowed. It is a trade-off between misclassification of the training samples and simplicity of the decision surface suitable for a general model.

In Equation (A2), *C* is the regularization factor that is optimized for the number of samples [42]. For a large value of *C*, the optimizer chooses a smaller-margin hyperplane if that hyperplane can classify all the training points correctly. Conversely, a small value of *C* causes the optimizer to look for a larger-margin separating hyperplane. The application of regularization improves the numerical stability and the universality errors for predicting unseen data.

(A2) $$\sum_{i}\varepsilon_{i}\leq C;\;y_{i}\left(\beta_{0}+\beta_{1}x_{i1}+\ldots \right)\geq M\left(1-\varepsilon_{i}\right),\;\varepsilon_{i}\geq 0$$

Four types of kernel functions *K(x_i_,x_j_)* in SVM were investigated in this study. They were linear, polynomial, radial basis function (RBF) and sigmoid kernel functions, expressed below in Equations (A3)–(A6), where *c*_0_ and *c*_1_ are the hyperparameters for the functions [48], and *γ* is the kernel coefficient, which defines how much influence a single training sample has. A large *γ* increases the area of influence of the support vectors but reduces the regularization for overfitting prevention, whereas a small *γ* constrains the model to capture the complexity of the data. The behavior of the model is very sensitive to the value of *γ*.

(A3) $$K\left(x_{i},\;x_{j}\right)=\varphi {\left(x_{i}\right)}^{T}\varphi \left(x_{j}\right)=\langle x_{i},x_{j}\rangle$$

(A4) $$K\left(x_{i},\;x_{j}\right)={\left[c_{0}+\gamma \langle x_{i},x_{j}\rangle \right]}^{c_{1}}$$

(A5) $$K\left(x_{i},\;x_{j}\right)=exp\left(-\gamma \|x_{i}-x_{j}\|{}^{2}\right)$$

(A6) $$K\left(x_{i},\;x_{j}\right)=tanh\left(c_{0}+\gamma \langle x_{i},x_{j}\rangle \right)$$

### Appendix A.2. k-Nearest Neighbors (kNN)

The *k*-nearest neighbors (*k*NN) algorithm is a non-parametric classification approach that classifies a point based on the majority class of the *k*-neighbors closest to the point. The average response of the *k*-closest points to *x* is given by Equation (A7).

(A7) $$f\left(x\right)=\frac{1}{k}\underset{i=1\ldots k}{\sum }y_{i}$$

The Euclidean distance *d*(*x_i_*,*y_i_*), expressed in Equation (A8), is usually adopted for calculating the distance [49].

(A8) $$d\left(x_{i},y_{i}\right)=\sqrt{\underset{i=1\ldots k}{\sum }{\left(x_{i}-y_{i}\right)}^{2}}$$

The neighbors closer to a query point have a greater influence than the neighbors that are farther away. Therefore, the predictions $\hat{y}$ can be made with a non-negative weight function to the neighbor distance *W*~*d_k_*^−1^, as shown in Equation (A9).

(A9) $$\hat{y}=\underset{i=1\ldots n}{\sum }W\left(x_{i},\;x_{j}\right)x_{i}$$

### Appendix A.3. Logistic Regression

A logistic regression algorithm is a linear classification model. The probabilities of the outcomes of a single trial are modelled using the logistic function exhibited in Equation (A10), where *x*_0_ is the *x* value of the sigmoid’s midpoint, and *k* is the logistic growth rate [50].

(A10) $$f\left(x\right)=\frac{1}{1+exp\left[-k\left(x-x_{0}\right)\right]}$$

The decision function is expressed in Equation (A11), where *w* is a coefficient vector.

(A11) $$f\left(x\right)=min_{w,c}\frac{1}{2}w^{T}w+C\underset{i=1\ldots n}{\sum }log\left(exp\left(-y_{i}\left(X_{i}^{T}w+c\right)\right)+1\right)$$

### Appendix A.4. Decision Tree (DT) and Random Forest (RF)

A decision tree (DT) is a non-parametric learning algorithm that partitions the data into subsets for classification [40]. The goal is to create the smallest possible tree (training model) that can predict the value of a target variable by learning simple decision rules. A tree can be seen as a piecewise constant approximation. The binary partitioning process continues until no further splits can be made so that the tree nodes are pure. The node purity can be measured by Gini impurity (GI) or by the information entropy (EI). GI measures the frequency at which any element of the dataset is mislabeled when it is randomly labeled. EI measures the disorder of the features with the target. A tree node is determined by minimizing the chosen index so that all the contained elements in the node are of one unique class. The GI and EI can be expressed by Equations (A12) and (A13), where *p_j_*^2^ is the probability of class *j*.

(A12) $$GI=1-\underset{j}{\sum }p_{j}^{2}$$

(A13) $$EI=-\underset{j}{\sum }p_{j}log_{2}p_{j}$$

Regularization can be done by confining the tree size, the tree’s maximum depth *D*, the minimum number of samples required to split an internal node *n_s_*, and the minimum number of samples required to be at a leaf node *n_r_*.

A random forest (RF) is a meta-estimator that fits several decision tree classifiers to various subsamples of the dataset. It is also known as a random decision forest (RDF) that uses the mode of the classification to improve the predictive accuracy and control the problem of over-fitting [51]. The number of trees in the forest is a hyperparameter to be tuned, in addition to those hyperparameters for a decision tree.

### Appendix A.5. Multilayer Perceptron Artificial Neural Network (MLP-ANN)

A multilayer perceptron artificial neural network (MLP-ANN) is a supervised learning algorithm that learns a function *f*(): *R^m^* → *R^o^* by training a dataset *R* with *m*-dimensional input and *o*-dimensional output. It can also learn a nonlinear function approximated for predicting the output. As ANNs do not have predefined assumptions, they have a low sensitivity to error term assumptions and high tolerance to noise. Therefore, an MLP-ANN can be used to examine the relationships in complex nonlinear datasets in the same way as conventional statistical techniques, but without many of the parametric restrictions about the nature of the data relationships [29]. The algorithm is described by Equation (A14), where *J* is the local gradient of function f concerning parameters *β*, *y* is independent variables and *δ* is the increment.

(A14) $$\left(J^{T}J+\lambda diag\left(J^{T}J\right)\right)\delta =J^{T}\left[y-f\left(B\right)\right]$$

The hyperparameters are adjusted for model performance. Hidden layer arrangement includes the number of hidden layers and the number of neurons in each hidden layer. The activation function of a neuron defines the output of that neuron given an input. Four activation functions (identity, logistic, *tanh* and rectified linear unit (ReLU)) used in this study are given in Equations (A15)–(A18).

(A15) $$f\left(x\right)=x$$

(A16) $$f\left(x\right)=\frac{1}{1+exp\left(-x\right)}$$

(A17) $$tanh\left(x\right)=\frac{exp\left(x\right)-exp\left(-x\right)}{exp\left(x\right)+exp\left(-x\right)}$$

(A18) $$f\left(x\right)=\left\{\begin{array}{cc} 0 & x\leq 0\\ x & x>0 \end{array}\right.$$

Moreover, iterative methods adopted for training the neural networks (weight optimization) can be specified. The L-BFGS type quasi-Newton method calculates the second derivative of the objective function and that leads to a more efficient descent direction [52]. Stochastic gradient descent (SGD), by using an estimate calculated from a randomly selected subset of the data rather than the entire dataset, optimizes an objective function with differentiable smoothness properties [53]. Adaptive moment estimation (Adam) is an algorithm for first-order gradient-based optimization of stochastic objective functions, based on adaptive estimates of lower-order moments [54].

Learning rate determines the weight updates. The default value for the constant learning rate is 0.001 for all iterative methods. Optional weights are available for the stochastic gradient descent solver. An “invscaling” weight gradually decreases the learning rate at each time step using an inverse scaling exponent to the time step, while an “adaptive” weight keeps the learning rate constant, as long as the training loss keeps decreasing. Dividing the current learning rate by 5 is generally adopted for the adaptive weight.

## Appendix B

**Table A1:** Configuration sets of the model hyperparameters for the inner layer architecture for the MLP-ANN classifier.

Legend	Activation	*C*	Learning Rate	Solver	Legend	Activation	*C*	Learning Rate	Solver
1	identity	0.0001	constant	Adam	31	relu	0.05	adaptive	SDG
2	logistic				32	*tanh*			
3	relu				33	identity	1		
4	*tanh*				34	logistic			
5	identity	0.05			35	relu			
6	logistic				36	*tanh*			
7	relu				37	identity	0.0001	constant	
8	*tanh*				38	logistic			
9	identity	1			39	relu			
10	logistic				40	*tanh*			
11	relu				41	identity	0.05		
12	*tanh*				42	logistic			
13	identity	0.0001		LBFGS	43	relu			
14	logistic				44	*tanh*			
15	relu				45	identity	1		
16	*tanh*				46	logistic			
17	identity	0.05			47	relu			
18	logistic				48	*tanh*			
19	relu				49	identity	0.0001	invscaling	
20	*tanh*				50	logistic			
21	identity	1			51	relu			
22	logistic				52	*tanh*			
23	relu				53	identity	0.05		
24	*tanh*				54	logistic			
25	identity	0.0001	adaptive	SDG	55	relu			
26	logistic				56	*tanh*			
27	relu				57	identity	1		
28	*tanh*				58	logistic			
29	identity	0.05			59	relu			
30	logistic				60	*tanh*			
